# Site Formation Processes and Hunter-Gatherers Use of Space in a Tropical Environment: A Geo-Ethnoarchaeological Approach from South India

**DOI:** 10.1371/journal.pone.0164185

**Published:** 2016-10-26

**Authors:** David E. Friesem, Noa Lavi, Marco Madella, P. Ajithprasad, Charles French

**Affiliations:** 1 McDonald Institute for Archaeological Research, University of Cambridge, Cambridge, United Kingdom; 2 Department of Anthropology, University of Haifa, Haifa, Israel; 3 Department of Humanities, CaSEs Research Group, Universitat Pompeu Fabra and IMF-CSIC, Barcelona, Spain; 4 ICREA, Pg. L. Companys 23, 08010, Barcelona, Spain; 5 Department of Archaeology and Ancient History, MS University of Baroda, Vadodara, Gujarat, India; 6 Department of Archaeology and Anthropology, University of Cambridge, Cambridge, United Kingdom; Max Planck Institute for the Science of Human History, GERMANY

## Abstract

Hunter-gatherer societies have distinct social perceptions and practices which are expressed in unique use of space and material deposition patterns. However, the identification of archaeological evidence associated with hunter-gatherer activity is often challenging, especially in tropical environments such as rainforests. We present an integrated study combining ethnoarchaeology and geoarchaeology in order to study archaeological site formation processes related to hunter-gatherers’ ways of living in tropical forests. Ethnographic data was collected from an habitation site of contemporary hunter-gatherers in the forests of South India, aimed at studying how everyday activities and way of living dictate patterns of material deposition. Ethnoarchaeological excavations of abandoned open-air sites and a rock-shelter of the same group located deep in the forests, involved field observations and sampling of sediments from the abandoned sites and the contemporary site. Laboratory analyses included geochemical analysis (i.e., FTIR, ICP-AES), phytolith concentration analysis and soil micromorphology. The results present a dynamic spatial deposition pattern of macroscopic, microscopic and chemical materials, which stem from the distinctive ways of living and use of space by hunter-gatherers. This study shows that post-depositional processes in tropical forests result in poor preservation of archaeological materials due to acidic conditions and intensive biological activity within the sediments. Yet, the multiple laboratory-based analyses were able to trace evidence for activity surfaces and their maintenance practices as well as localized concentrations of activity remains such as the use of plants, metals, hearths and construction materials.

## Introduction

Compared to agriculturalists, hunter-gatherer societies have more ephemeral site structures also due to the use of less durable materials in their activities (e.g., [1–4]). From an archaeological perspective (and taphonomical), the identification of settlement and materials related to hunter-gatherers is therefore challenging. This difficulty is amplified when addressing open and semi-open-air sites located in tropical forests. It is generally assumed that due to the environmental conditions of the tropics many materials remains from human activity do not preserve [5, 6]. In comparison to arid, semi-arid and temperate environments, site formation processes in tropical forests (specifically open-air-sites) have been studied in less detail. Few geoarchaeological studies in Southeast Asia tropical forests focused on identification of anthropogenic remains in caves and rock shelters (e.g., [7–9]) and open-air sites (e.g., [10]), using micromorphology. In this study we examine the archaeological site formation processes and use of space of hunter-gatherers in tropical forests in various settlements (open-air sites and a rock-shelter) by the combined use of different proxies. To do so we embraced an integrated approach to investigate the formation of the archaeological record at microscopic and submicroscopic level using elemental and mineralogical analysis, phytolith quantification and micromorphology.

Studying archaeological site formation processes involves the investigation of the anthropogenic factors—the processes driven by human activity forming and shaping the archaeological material record, and the natural and environmental factors—the geochemical processes altering or preserving the materials after their deposition [11]. Following the transformations of archaeological materials starting from the initial deposition connected to human activities to the post-depositional processes occurring pre- and post-abandonment is rarely possible in an archaeological setting. A geo-ethnoarchaeological approach, in recently abandoned settlements, however, enables direct connections to be established between human behaviour and the resulting material deposition patterns. Furthermore, the analyses of related sediments from these sites can be usefully used to monitor the early post-depositional processes [12].

Although hunter-gatherer use of space has previously been studied through ethnoarchaeology at the macro-level (e.g., [1, 2, 4, 13, 14], see also [15] for more examples), we suggest an approach with a micro- and sub-microscopic perspective, also adding an investigation of the archaeological formation processes evident in a tropical setting. This involves the integration of: 1) ethnographic work with a contemporary hunter-gatherer group in a tropical forest of south India, 2) targeted ethnoarchaeological fieldwork including excavation of recently abandoned open-air sites and a rock-shelter, and 3) geoarchaeological laboratory-based analyses of the sediment sequences.

The overall aim of this paper is to study the anthropogenic and natural site formation processes associated with hunter-gatherers sites in tropical forests. Specific objectives of this research are twofold: 1) the study of material deposition patterns at microscopic scale that result from hunter-gatherer activity and 2) the investigation of natural post-depositional processes in tropical forests. The current sites are used as a pilot study to understand the use of space within hunter-gatherers habitation site as manifested by a set of different proxies (chemical and mineralogical analysis, phytolith quantification and micromorphology). There is no suggestion that diverse contemporary hunter-gatherer groups are culturally homogenous or that these groups represent a relic of prehistoric hunter-gatherers. We embrace here an approach which examines how specific social practices, mainly mobility and immediacy, are expressed in the spatial distribution of microscopic and elemental signatures, and how they preserve after abandonment in a tropical environment.

### The Environmental and Cultural Setting

**Fig 1:**
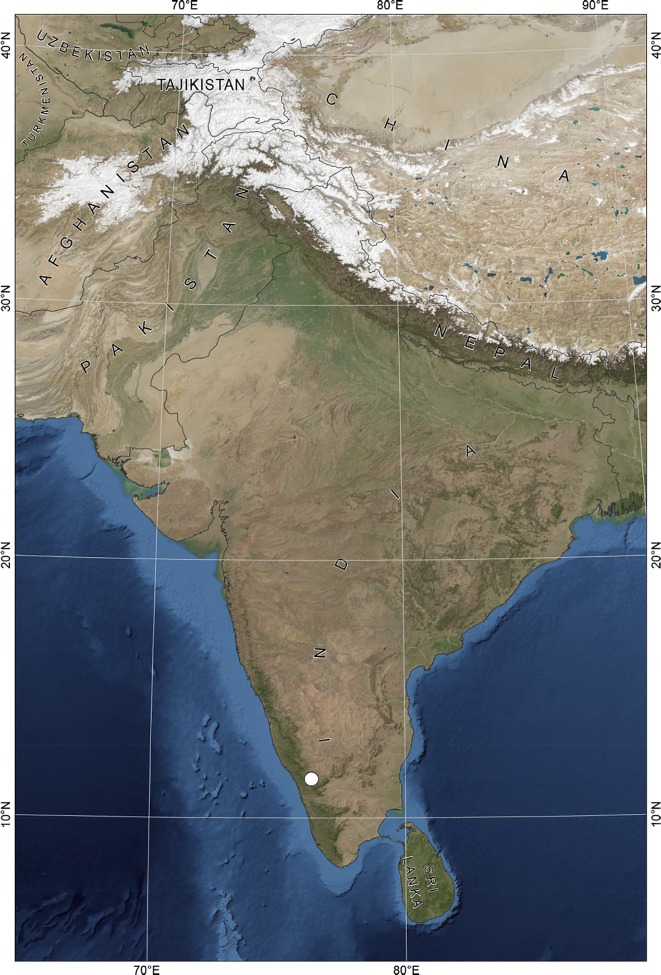
Location of field. Map showing the location of the fieldwork in the mountain ridge of the Western Ghats in South India in white circle. The Blue Marble Next Generation data is courtesy of Reto Stockli (NASA/GSFC) and NASA's Earth Observatory. NASA/Goddard Space Flight Center Scientific Visualization Studio. The country data is taken from the: CIA World DataBank II.

The study area is located in the forested hills of the Western Ghats mountain ridge in South India (Fig 1). This forested area forms part of the Nilgiri Biosphere Reserve (NBR) (10° 45’N to 12° N and 76° E to 77° 5’ E) lying between the states of Karnataka, Kerala and Tamil Nadu. Sites were located at altitude of between 700 and 900 meters above sea level. The natural vegetation of the NBR ranges from wet evergreen tropical forests to thorn forests [16], and it varies depending on the geography and altitude. The temperature ranges from 17 to 37°C with average annual precipitation of 2600mm. Most precipitation falls during the monsoon season, from June to September [17]. The geology of the area is characterised by rocks rich in quartz, orthopyroxene and magnetite such as charnockites and enderbites [18].

Fieldwork was conducted among a Nayaka hunter-gatherer group. The Nayaka were classified as immediate-return forest dweller hunter-gatherers by Bird-David (see selected publications [19–22]). Bird-David drew on the Nayaka social perceptions and ways of living to demonstrate how the Nayaka and other similar hunter-gatherers groups maintain social relations and perceive themselves and their environment through living-together and sharing (e.g., [21, 22]). The Nayaka used to live in small hamlets located deep in the forest. Each hamlet consisted of several houses scattered over a relative short distance (i.e. between a few meters to an hundred meters). Houses were generally built from grass, bamboo and in some cases mud-bricks. Houses were never completely sealed allowing visibility between the houses. Houses were usually square with each one having an open terrace outdoor at its front where the vast majority of activities took place. Such activities included: processing and consumption of food, production and maintenance of metal and wooden tools, use of fire, clearing and sweeping of the terraces and house floors, and socializing (note: a detailed description of Nayaka domestic activity and use of space will be published elsewhere by Friesem and Lavi [23]; for more detail account on the Nayaka houses see [3, 24]).

More recent work by Lavi (e.g., [24, 25]) explored the current Nayaka in the light of contemporary socio-economical changes happening in their territory (i.e. increasing intervention of development agents, deforestation, enforcement of new laws and restrictions, and increased engagement of Nayaka people with outsiders). Lavi’s study argues that, though one can easily point out significant changes in many aspects of the Nayaka's lives, the ways in which they perceive these changes and engage with them reflect a great deal of continuity in their perceptions, values and ideas. An ethnoarchaeological study by Friesem and Lavi [23] examined material deposition patterns resulting from the Nayaka way of living. The authors associated everyday practices, such as immediacy, mobility and sharing of spaces, things and actions, with distinct patterns of the use of space. An interesting phenomenon observed in the study was that of the deposition of materials related to activities not associated to specific activity areas (as opposed to neighboring farming communities). Indeed, the deposition of materials indicated how these activities were conducted in various areas according to the group’s social dynamics and the *ad hoc* relationships between the people at the site.

Based on these observations, the current geo-ethnoarchaeological study was carried out in order to test the preservation of anthropogenic activity markers related to hunter-gatherers way of living and to evaluate the post-depositional effect and processes on these markers in a tropical environment.

## Materials and Methods

### Ethnography

Ethnographic study was carried out by Lavi and Friesem in a contemporary settlement located at the fringe of the forest. Permits for fieldwork were obtain through affiliation with the MS University of Baroda and the Spain-India collaborative NoGAP project. Observations and interviews were made through numerous visits to the same Nayaka site in 2010, 2012 and 2014, each time for a period of two, four and six months respectively. The informants have given their oral consent for the work and all the ethnographic data is reported anonymously. The ethnographic data supplied valuable information regarding peoples’ use of space and the material deposition patterns related to these tropical forest hunter-gatherers. In addition, field observations, excavations and sediment sampling were conducted in several other localities in the same forest area. Those localities were old sites, including open-air sites and a rock-shelter, abandoned ca. 20–30 years ago by the same group of people. These abandoned sites were located deeper in the forest and were recognized and introduced to us by the elder Nayaka people in the village where the ethnographic work took place.

### Excavation and sampling

**Fig 2:**
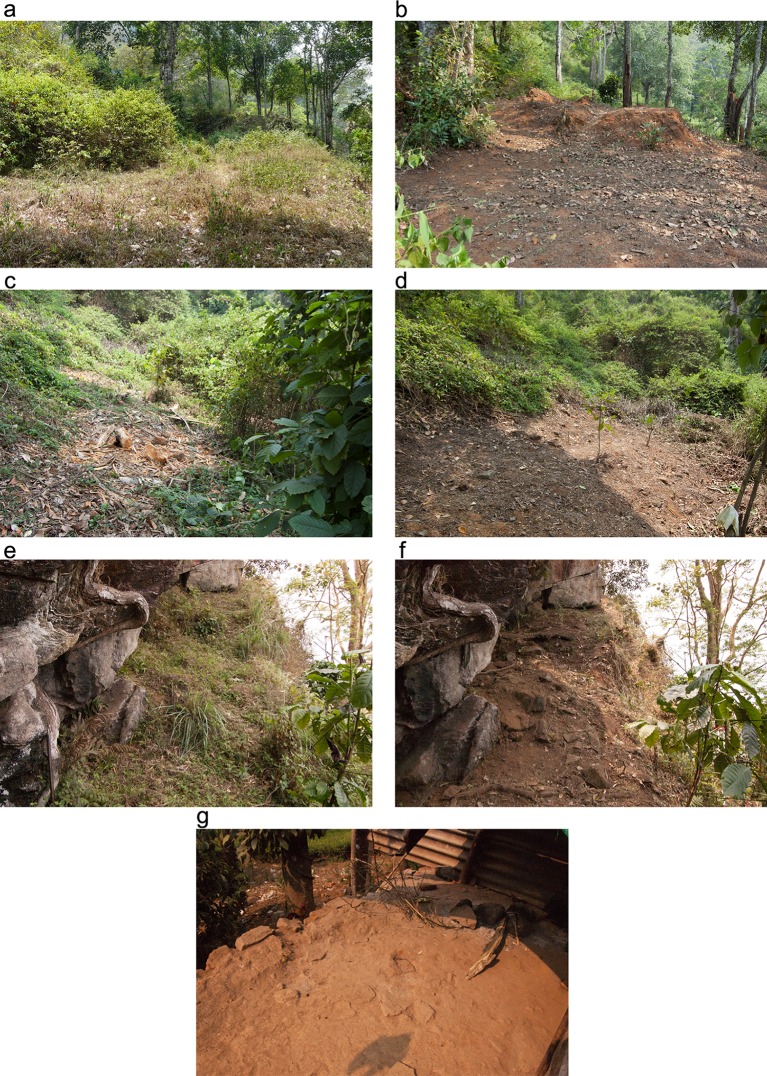
The localities sampled during fieldwork. (a) Open-air site 1 before and (b) after clearing the forest vegetation. Note in the background the earth mound—formed following the decay of a house—located on a flat terrace ending with a steep slope at the far right end of the picture. The width of both pictures is ca. 10m; (c) Open-air site 2 before and (d) after clearing of the forest vegetation. Note how the site formed a flat terrace within a steep forest hill slope. The width of the terrace is ca. 5m; (e) The rock-shelter before and (f) after clearing of the forest vegetation. The site is inclined toward the far end of the picture. Note how narrow the rock-shelter ends on the right of a very steep slope. The width of the front of the picture is ca. 3m; (g) The contemporary site presenting the exterior terrace ending at the back of the picture in a slope. An active hearth is seen in the right part of the picture. The middle of the picture shows the location of a recently abandoned hearth, noticeable by the greyish colour of the terrace surface. The width of the picture is ca. 2m.

The research included small-scale excavation and surface sampling from four different localities. The first two localities–open-air site 1 (OA1 = ca.160m^2^) and open-air site 2 (OA2 = ca.90m^2^) were part of one settlement abandoned ca. 30 years ago (Fig 2A–2D). Each locality comprises house debris and an external activity terrace. The third locality was a rock-shelter (RS = ca.45m^2^) abandoned ca. 20 years ago (Fig 2E and 2F). All localities were mapped and divided into a 1x1m grid. Trenches of 1x1m were dug to a depth of between 30 and 50cm, a few centimeters below the sterile palaeosol layer. In the terrace of OA1 a long trench measuring 1x6m was excavated. Sediments were systematically sampled from the gridded surface and from columns taken from the profiles. Selective sampling was also employed on features of interest when a specific question arose in the field [26]. The fourth locality sampled was the contemporary site (CS)—where the people among whom the ethnographic study was conducted live today. Sediments were collected at 60cm intervals sampling from three small trenches, including the surface sediment (Fig 2G). Control sediments (i.e., regional sediments) were sampled outside the sites, a few hundred meters away, in order to obtain an environmental background with no anthropogenic signature. The control samples were then used as a baseline to trace human impact on the sediments sampled from the sites. Bulk samples were registered with sequential numbers and put in plastic bags using a metal spoon. Undisturbed blocks for micromorphological analysis were sampled based on contextual considerations.

### Elemental Analysis using Inductively Coupled Plasma-Atomic Emission Spectrometry (ICP-AES)

Multi-element analysis was carried out by using Inductively Coupled Plasma-Atomic Emission Spectrometry (ICP-AES) to provide quantitative data on the chemical elements. Representative bulk sediment samples (n = 77) were sent to ALS Laboratory Group in Seville (Spain), using method ME-41 ICP, where they were pre-treated with aqua-regia digestion for ICP-AES to measure the concentration of 35 main elements from Aluminum to zinc [27]. Elements with values below the reliable instrument detection limits (IDL) were excluded (samples values and analyses are presented in S1 Table).

### Mineralogy using Fourier-Transform Infra-red (FTIR) spectroscopy

All the bulk sediment samples (n = 110) were analyzed using Fourier-Transform Infrared (FTIR) spectroscopy in order to identify the mineral and organic components for each sample. The spectra were collected using the KBr method between 4000 and 250cm^-1^, at 4cm^-1^ resolution using a Thermo Nicolet 380 spectrometer and interpreted using an internal library of infrared spectra of archaeological materials at the Kimmel Center for Archaeological Sciences at the Weizmann Institute of Science [28].

### Phytolith Quantification

Phytoliths were extracted from representative bulk sediment samples (n = 59) following the procedure of Katz et al. [29]. Phytoliths were counted using a petrographic microscope (Zeiss Axio) at x200 magnification. The concentrations of phytoliths per gram sediment was calculated following the equation described by Katz et al. [29] (Phytolith concentrations for each sample are presented in S2 Table).

### Thin Sections or Sediment Microscopy

Undisturbed monolith sediment blocks (n = 5) were sampled using jackets made of Plaster of Paris. The blocks were dried in an oven at 30°C and then impregnated using a 9:1 mixture of polyester resin with acetone and 1% v:v MEKP. Pre-cut sample slices (2.6x5.5 inch) were ground to 30μm thickness thin sections following the method described by Murphy [30]. Thin sections were first studied at a scale of 1:1, scanned using a flatbed scanner, and then analyzed with a petrographic microscope at magnifications ranging from x4 to x250 with plane-polarized light (PPL) and cross-polarized light (XPL). Micromorphological descriptions employ the terminology of Bullock et al. [31] and Stoops [32].

## Field Observations

**Fig 3:**
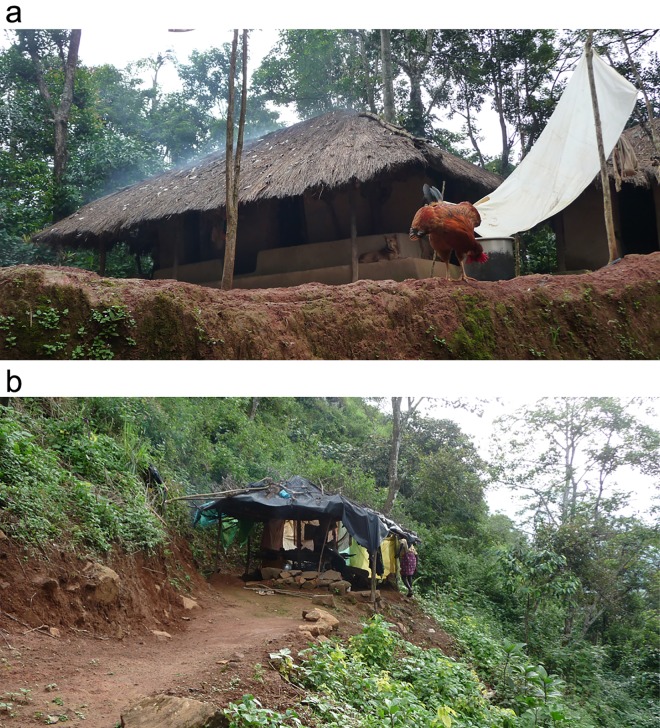
Typical Nayaka houses. (a) A house located on a flat terrace which ends in a steep slope. The house is built on a platform made of stones and mud. Walls are partially made of mudbricks, branches, bamboo and grass and in some cases plastered with mud. The roof is made from branches covered with grasses. Note how the roof extends the house outline leaving the edges of the platform to be used as an open, yet roofed, veranda; (b) A typical open house. The house lack walls and while the structure is made of branches the roof was made of plastic sheets. Note how the house is located on a terrace made by incision in the forest steep hill slope.

The ethnographic observations conducted on contemporary site recorded how people’s activity resulted in the deposition of materials and how post-depositional behaviour influenced the preservation of these materials. Houses were mostly built from plant material such as bamboo, wood and grasses, but in some cases mud-bricks were also used (Fig 3A). A typical house is set on a flat terrace that is created through an incision in the hill-slope and soil removal. Most houses are built on top of a mud-plastered stone platform and they have the roof protruding away from the house outline creating a covered veranda on part of the platform (Fig 3A). Some of the houses are lacking walls, being completely open (Fig 3B). The vast majority of domestic activities were performed outside on the terrace and the indoor was used mostly for sleeping during cold and windy nights. Activities were carried out in different locations but without specific areas assigned to specific activities. Indeed, activities changed location according to the immediate and dynamic social needs, displaying a heterogeneous material deposition pattern.

**Fig 4:**
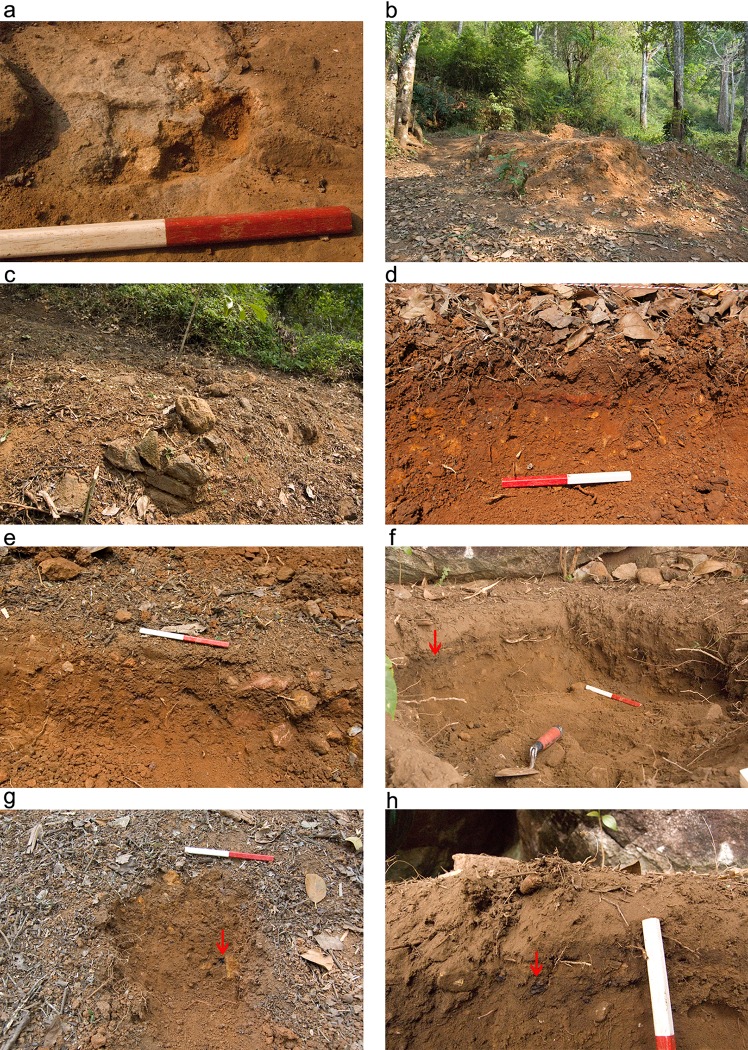
Field observation and macroscopic finds. (a) The ashy layer sampled from the location of a recently abandoned hearth in the contemporary living terrace. Note that under the millimeter thick ash layer on the surface a white fragmented material is revealed which might represents a bone fragment. The red part of the scale bar is 10cm; (b) Earth mound in open-air site 1 formed following the degradation of a house partially made of mudbricks. Note the remains of the rectangular bricks on the right side of the mound. The width of the picture is ca. 7m; (c) The remains of a small wall found at open-air site 2; (d) A profile revealed by trenching the terrace (sq. OA1-F5) of open-air site 1 showing a lower brown-reddish layer with yellow and red rock fragments overlain by a brown layer covered by leaves in various stages of decomposition. Scale bar is 20cm; (e) A sediment profile revealed in open-air site 2 (sq. OA2-D5) showing a bioturbated brown sediment with rock fragments. Note the organic-rich topsoil covered by leaves in various stages of decomposition. Scale bar is 20cm; (f) A profile exposed the rock-shelter (sq. RS-B9). The sedimentary sequence presents a lower dark brown sediment overlain by a thin black layer (arrow) below fine grey-brown topsoil. Scale bar is 20cm; (g) The profile revealed in the trench of open-air site 1 slope (sq. OA1-H11). Note the lack of any layering and the black charcoal particles (arrow). Scale bar is 20cm; (h) Close-up photograph of the thin blackish layer in the rock shelter (sq. RS-B9) showing fragmented black charcoal particles (arrow). The white part of the scale bar is 10cm.

The processing and consumption of food resulted in the deposition of small fragments of bones and the remains of vegetables, fruits, oils and other liquids. Today plastic is part of the deposited waste, coming from food wrapping of items purchased in the markets. Maintenance of steel machetes and the production of timber tools resulted in the deposition of small chips of vegetal matter and metals. Hearths generally were of an ephemeral nature as they were often abandoned after a few days use, and a new fire started in another location on the terrace. Hearths used small amounts fuel, mostly wood, that, nevertheless produced sufficient amounts of ash and charcoal to be deposited on the terrace surface. Yet, the most significant activity in terms of material deposition patterns was the sweeping of the terraces, which was conducted on a daily basis. Sweeping resulted in the clearing and disposal of the waste materials that were normally re-deposited over the edge of the terrace. This process created small cumulative middens at the edge of the terraces on the hill-slope. The middens were actually the only designated areas where material were repeatedly deposited. Macroscopically, contemporary terrace surfaces look relatively clean of any activity remains except for some traces of ash in the location of recently abandoned hearths (Fig 4A). Following trampling, monsoon rains or after few days of abandonment, those few visible traces of ashes were no longer observed and clear evidence of waste were macroscopically observed only at the midden areas.

The abandoned sites, which were the main focus of this study, were covered with dense forest vegetation before excavations began (Fig 2A, 2C and 2E). It would have been impossible for us to locate them without the elder informants. The only observable anthropogenic features in these open-air sites were the relative flat surfaces indicating the abandoned terraces and the incision into the forest hill-slope made to construct the terraces and houses. Following the clearing of the forest vegetation (Fig 2B, 2D and 2F), we observed a small mound of debris as the remains of a partly earthen structure in open-air Site 1 and some stones from wall remains in open-air Site 2 (Fig 4B and 4C respectively).

During trenching of the sites some layering appeared in the excavation profiles but could not be interpreted as an activity surface as no artefacts were exposed during the excavation. The trench profiles revealed some variation. Open-air Site 1 had a lower brown-reddish layer, in some cases with rock fragments, and a brown to light brown sediment on top of it (Fig 4D). Open-air Site 2 showed a brown sediment underlying an organic-rich topsoil with plant material in different stages of decomposition (Fig 4E). The rock-shelter on the other hand presented some layering between a fine grey-brownish topsoil sediment and a lower dark brown to almost blackish sediment (Fig 4F).

The only macroscopic materials which could be associated with human activity were small fragments of charcoal found on the slope of open-air Site 1 (Fig 4G). This finding correlates well with the ethnographic data. The terrace surface has no remains due to sweeping and cleaning of the hearths, while the only place where occupation deposits were clearly accumulating was on the slopes in the middens. In the rock-shelter a thin fragmented layer of charcoal (ca. 1–2cm) was found in sq. RS-B9 (Fig 4H). This might suggest the preservation *in situ* of a hearth, which might have been used just before the abandonment of the site.

## Results and Discussion

### Elemental characterization via ICP-AES

**Fig 5:**
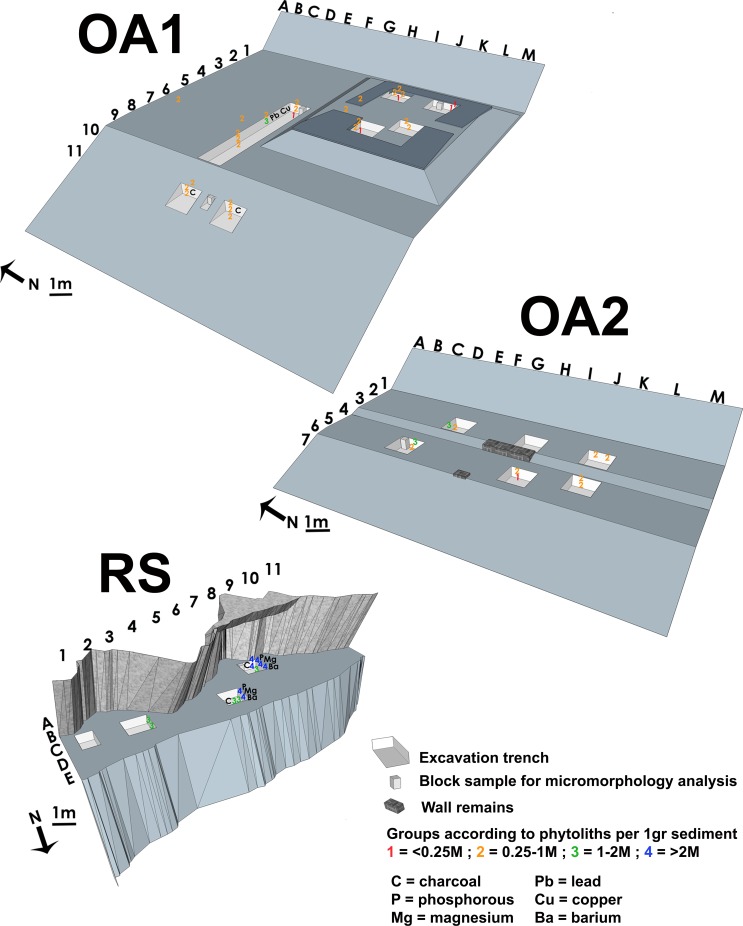
Schematic illustration of the excavated sites presenting the location of representative samples with concentrations of microscopic materials and chemical signatures. (a) Open-air site 1 (OA1) showing the presence of charcoal only on the slope. Note the elevated concentrations of heavy metals (Cu and Pb) and phytoliths found at the same middle layer of the terrace trench profile; (b) Open-air site 2 (OA2) showing elevated concentrations of phytoliths outside the remains of the walls and only at the upper layers in close proximity to the surface; (c) The rock-shelter (RS) showing relatively high amounts of phytoliths and elements associated with human activity (P, Mg, Ba). Note how the western part of the site presents the highest amounts of P, Mg, Ba and phytoltihs among the RS samples. Elevated concentrations are especially associated with the upper layers in close proximity to the surface. In addition charcoal remains were found in sq. C7 and a thin charcoal horizon was found in the middle layer of sq. B9 southern profile.

Geochemical analysis of archaeological sediments is being broadly used for tracing residues of human activity [11, 33]. Representative samples from each locality were analyzed and comparisons were made according to certain elements, which are considered associated with human activity (Fig 5 and S1 Table). Various ethnoarchaeological studies have demonstrated how elevated concentrations of phosphorus (P), magnesium (Mg) and potassium (K) are found in areas of food processing (e.g., [34–38]). In addition, barium (Ba), calcium (Ca), copper (Cu), sodium (Na), lead (Pb), strontium (Sr) and zinc (Zn) have been identified as chemical markers of intensive anthropogenic activity [39–41].

**Fig 6:**
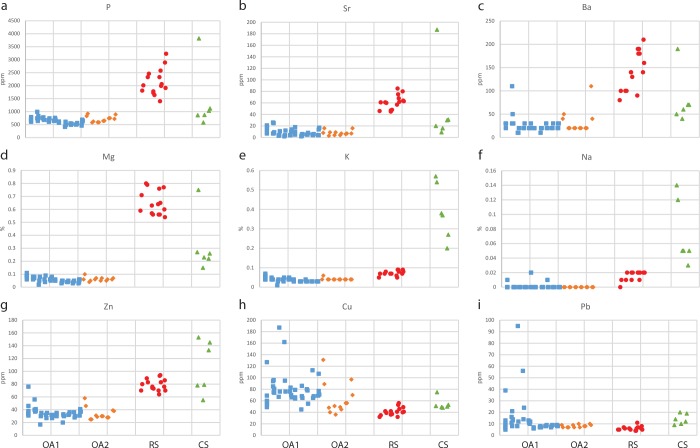
Plots showing the concentrations of selected elements. OA1 = open-air site 1 (blue squares); OA2 = Open-air site 2 (orange diamonds); RS = rock-shelter (red circles); CS = contemporary site (green triangles). (a) Concentrations of phosphorous (P) in parts per millions (ppm); (b) Concentrations of strontium (Sr) in ppm; (c) Concentrations of barium (Ba) in ppm; (d) Concentrations of magnesium (Mg) in percentages; (e) Concentrations of potassium (K) in percentage; (f) Concentrations of sodium (Na) in percentages; (g) Concentrations of zinc (Zn) in ppm; (h) Concentrations of copper (Cu) in ppm; (i) Concentrations of lead (Pb) in ppm.

The entire set of results obtained from the geochemical analysis, including 35 elements, is presented in S1 Table. In Fig 6 we show a comparison across the different localities for elements which presented a significant difference between the samples. The highest concentrations of P, Mg, Ba, K, Na, Sr and Zn were found in the fine grey sediment sample (CS-101) collected from the contemporary site terrace where a hearth was recently abandoned (Fig 4A). It is also the only sample which exhibited calcite and apatite minerals. The elemental analysis correlates very well with the ethnographic data and field observations of this context associated with the deposition of wood ash, food processing remains and the indications of the consumption of food taking place around the hearth (e.g., [34, 36–38, 42]). Yet, beside this exceptional sample, the other samples from the contemporary site terrace did not exhibit such high concentrations, although similar activities were conducted throughout the terrace. This might hint at relatively rapid post-depositional processes occurring in such an environment which do not favor the preservation of anthropogenic signatures.

The concentrations of P, Sr, Ba, and Mg (Fig 6A–6D) were higher in the rock-shelter samples compared to other samples from the contemporary site and all the samples from the open-air sites. Within the rock-shelter samples, higher concentration of P, Sr and Ba were associated with the upper layers in sq. RS-C7 and B9 (see Fig 5 and the right part of the rock-shelter cluster in Fig 6A–6C). The higher presence of those elements in the rock-shelter could be attributed to deposition of organic matter and the natural protection from the weather that the rock-shelter is providing.

The concentrations of K, Na and Zn were higher in the contemporary site samples than in the rock-shelter samples, while the sediments of the two open-air sites exhibited even lower concentrations (Fig 6E–6G). To the naked eye, the area sampled in the contemporary site did not appear to possess accumulations of organic matter or other activity remains (Fig 2G) but the ethnographic observations reveal that the Nayaka were active across the entire terrace surface. The samples from the contemporary living terrace surface demonstrate that while macroscopic evidence for human activity is scarce, the microscopic anthropogenic signatures are significant. Nevertheless, these signatures seem to decrease over time due to environmental post-depositional processes. In addition, the rock-shelter, partially protected from the elements, may present a better state of preservation due to its physical setting compared to the open-air sites.

Significant concentrations of Cu and Pb (Fig 6H and 6I) were found in two samples (OA1-31 and OA1-35) which were collected from open-air site 1, both from the terrace trench from the middle and lower part of squares OA1-F4 and F7, respectively (Fig 5). High concentrations of Cu and Pb in the sediment samples could be associated with contamination of heavy metals due to the sharpening of machetes [38]. During the ethnographic work, machete sharpening was routinely conducted on the terrace by powdering rock fragments (probably containing quartz crystals) and grinding the knives on the gritted surface of a wood branch, which reproduced a sharpening stone. It is therefore suggested that the traces of heavy metals mark the location of a buried terrace surface where activity such as machete sharpening was conducted in the past. There is also the possibility that such a chemical signature would not be found in other localities simply due to the limited number of samples collected.

### Mineralogical characterization via FTIR spectroscopy

**Fig 7:**
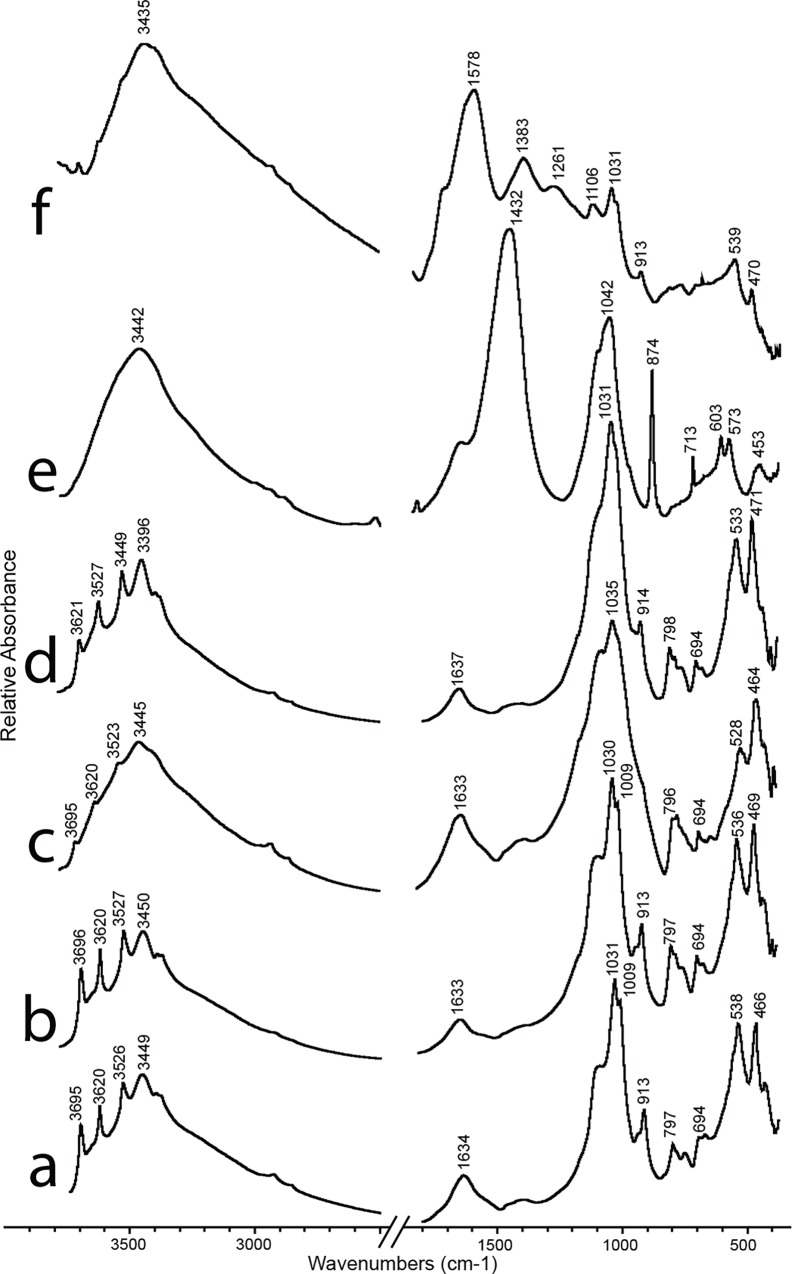
Representative Infrared spectra from the various localities. (a) Spectrum of a sediment from open-air site 1 showing kaolinite as the major component with indicative absorbance bands at 1031, 1009, 913, 694, 538, 466, 3449, 3526, 3620 and 3695cm-1. In addition the absorbance band at 797cm-1 may indicate the presence of quartz while hematite also shares prominent absorbance bands at 540 and 470cm-1. The broad absorbance band at 1634cm-1 is associated with organic matter; (b) Representative spectrum of a sediment sample from open-air site 2 showing high similarity to the sediments in open-air site 1; (c) Representative sediment sample spectrum from the rock-shelter. Note the shift toward higher wavenumbers of the main silicate absorbance band positioned at 1035cm-1, the reduction in the absorbance bands at 1009 and 913cm-1 (which appear as small shoulders) and the absorbance bands at 3695-3445cm-1. In addition, the absorbance of the band in 1633cm-1 increased; (d) Representative spectrum from the samples collected from the contemporary terrace. The spectrum shows high similarity to the sediments from the open-air sites; (e) The ashy layer collected from the recently abandoned hearth in the contemporary living terrace (CS-101). The spectrum showing calcite as the major component with indicative absorbance bands at 1432, 874, 713cm-1. In addition, the absorbance bands at 1042cm-1 and the doublet at 603 and 573cm-1 indicate the presence of carbonate fluorapatite (i.e., francolite); (f) Representative spectrum of the black particles found in the slope of open-air site 1 and in the rock-shelter. The spectrum is indicative of charcoal with absorbance bands at 1578, 1383, 1261, 1106 and the broad band at 3345cm-1. In addition to charcoal a minor amount of clay is also present.

The mineralogical composition of the sediment from the two open-air sites was similar. Kaolinite was the major component, along with quartz, iron oxides and organic matter (Fig 7A and 7B). The samples from the rock-shelter exhibited generally similar composition to the open-air sites but with some variation in silicates. Some absorbance bands were less prominent (i.e. 1009, 914cm^-1^ and the bands in the O-H region of 3695-3445cm^-1^) and in most samples the main silicate absorbance band shifted to 1035cm^-1^ and it was wider (Fig 7C). Although shifts in the main silicate absorbance band position can also result from clay alteration due to exposure to high temperatures [43], the interpretation of burning events based only on the position of the main silicate absorbance band is problematic [44, 45]. The presence, even if fairly minor, of absorbance bands and/or shoulders at 3695, 3620, 3523, 3445 and 914cm^-1^, indicates a more complex story of deposition and site formation processes which do not necessarily indicate burning. In addition, compared to the open-air sites, the rock-shelter sediments had a more prominent absorbance band at 1633cm^-1^ that can be associated with higher amounts of organic matter (Fig 7C).

The infrared spectra of the samples collected from the terrace of the contemporary site showed similarity to the ones from the open-air sites (Fig 7D). The grey fine sediment sample (CS-101) collected from the terrace surface, where a hearth was recently abandoned (Fig 4A), was mainly composed of calcite mixed with carbonate hydroxylapatite (Fig 7E). Calcite is the major mineral component of wood ash [46]. But, it is worth mentioning that this was the only sample in all localities that exhibited calcite. The lack of calcite preservation in the other samples from the contemporary living terrace—where hearths were also previously placed–indicates rapid dissolution probably due to intensive precipitation during monsoons rains and an acidic environment with the pH lower than 6 (e.g., [47]). The position and relative absorbance of the apatite mineral, with its main absorbance band at 1042cm^-1^ and with the 603cm^-1^ band higher than the 573cm^-1^, suggests the presence of carbonate fluorapatite, also termed francolite [28]. Carbonate fluorapatite has been reported in archaeological sites as a marker of bone diagenesis [48]. It is therefore possible that water rich in fluoride reacted with the ash or some bone fragments mixed with the ash. Under the acidic conditions with pH below 6, carbonate hydroxylapatite dissolves while authigenic carbonate flourapatite is more stable [46]. Such rapid formation of authigenic mineral and the dissolution of calcite indicate the quick and strong post-depositional processes in these tropical environments (e.g., [8]).

Black particles extracted from several samples presented absorbance bands indicative of charcoal (Fig 7F). By using infrared spectroscopy we could identify more samples containing micro-charcoals which we could not observe on the site during the excavation. Micro-charcoals were only found in samples from the slope of open-air Site 1 (sq. OA1-F11 and H11) and from two trenches of the rock-shelter (sq. RS-C7 and B9) (Fig 5). While the ethnographic data showed the routine use of fire and consumption of meat, wood charcoal was identified mostly at microscopic scale. Except for one sample of the contemporary site, a recently abandoned hearth, no other traces of ash (calcite) or of bone (carbonate hydroxylapatite) were observed in any of the infrared spectra. This implies that the weakly acidic environmental conditions and the abundance of water caused the dissolution of carbonates [28]. Kourampas et al. [7] and Lewis [8] report similar patterns from Kitulgala Beli-lena rock-shelter in Sri Lanka and Tabon Cave in the Philippines respectively, where a lot of micro-charcoals were identified as well as some phytoliths but almost no ash and bones at a microscopic scale.

### Phytolith analysis

**Fig 8:**
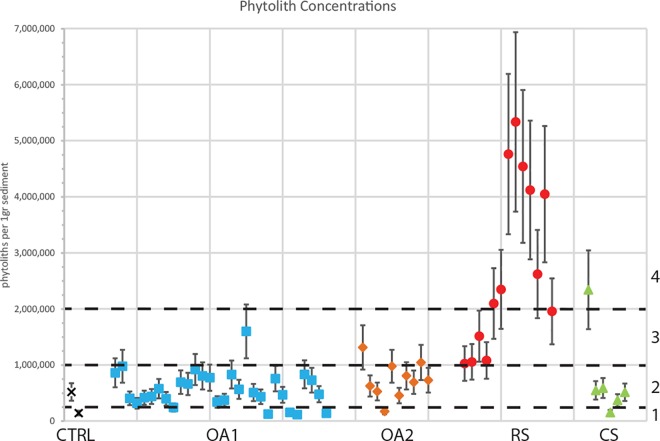
Plot showing the concentrations of phytoliths per 1g of sediment. CTRL = control sediment samples (black X); OA1 = open-air site 1 (blue squares); OA2 = Open-air site 2 (orange diamonds); RS = rock-shelter (red circles); CS = contemporary site (green triangles). The dashed lines indicate the border between the four groups (marked in numbers on the right side) according to the phytolith concentrations. (1) Samples with less than 0.25M phytoliths per 1g sediment, including buried soil layers and degraded earthen construction materials found in the mound in OA1; (2) Samples with 0.25-1M phytoliths per 1g sediment, including the control topsoil sample, surface samples from the terrace of CS and most of the samples from OA1 and OA2; (3) Samples with 1–2M phytoliths per 1g sediment, including one sample from OA1 (OA1-43 collected from the middle part of the terrace trench), two samples from OA2 (OA2-62 and OA2-76 collected from the upper parts of sq. D5 and E3 profiles), and samples from the eastern part of the rock-shelter (sq. C3 and C7 north profile). This group of samples probably indicate the deposition of phytoliths due to human activity; (4) Samples with more than 2M phytoliths per 1g sediment, including the ashy sample from CS (CS-101) that represent ash deposition from a recent hearth abandonment, and samples from the western part of the rock-shelter (in the upper and middle part of the profiles of Sq. C7—west profile only—and B9), which may indicate a layer of collapsed lean-to thatch roof.

High concentrations of phytoliths in sediments can be used as a reliable indicator of human activities associated with the exploitation of plants (e.g., [29, 45, 49–54]). The goal of this study was to detect possible diversity in phytolith concentrations that could be interpreted as anthropogenic signature of hunter-gatherer frequentation and spatial distribution of activities at the site. This is an important first step development in understanding phytolith deposition in tropical open and semi-open-air sites. We considered the taxonomy of phytoliths at this stage less significant in our study for two reasons: 1) production of phytoliths in tropical forests is often dominated by arboreal dicotiledon, and 2) there is currently no detailed reference collection for the area to justify tentative taxonomic identification. Phytolith concentrations were quantified in representative sediment samples from all the sites and the results are presented in S2 Table and Fig 8.

Two phytolith control samples were collected from a profile outside the range of human activities. The sample collected from the topsoil showed higher concentrations of phytoliths (520,000/g of sediment) than the sample collected below the topsoil (144,000/g of sediment) (Fig 8 and S2 Table).

The samples collected from the sites were divided into four groups according to their concentrations of phytoliths (Fig 8). The first group includes samples with less than 250,000/g of phytoliths. These samples are associated with buried soil layers from the lower parts of the trench profiles in open-air sites 1 and 2 and the contemporary site. In addition, sediments from the decayed mud-house mound in open-air site 1 also showed a low concentrations of phytoliths (Fig 5 and S2 Table). The similarity of phytolith concentrations between the sediments from the collapsed house and the buried soil seems to indicate the use of this buried, rich in clay, as raw material for the construction of the house. The Nayaka probably removed the uppermost layer of the local soil in order to reach the clay-rich B horizon to prepare sun-dried mud-bricks and for wall plastering. After site abandonment, the degradation process of the mud-house involved the breakdown of the earthen construction material and its displacement away from the original structure to form a mound (see [45, 55] for detailed description on mud structure degradation and the formation of mounds).

The second group of samples was attributed to sediments with phytolith concentrations ranging between 250,000 and 1M/g of sediment. This group includes the majority of the sediments sampled in the open-air and contemporary sites (Fig 8). The control topsoil sample (with 520,000/g sediment) also forms part of this group, as well as the samples from the surface of the contemporary terrace (ranging between 370,000–591,000/g of sediment). This suggest that Nayaka activities do not necessarily result in noticeable enrichment of the forest phytolith input (Fig 5 and S2 Table). This is confirmed by ethnographic observations showing that Nayaka hunter-gatherers use of space in connection to daily chores does not result in a significant accumulation of activity residues, even within the foci of activity areas. Waste areas, where materials are being re-deposited as part of cleaning and site maintenance practices, present similar amount of phytolith but present higher accumulation of other occupation residues [4, 14, 23].

The third group of samples comprised sediments with phytolith concentrations higher than the control samples, ranging from 1M to 2M phytoliths/g sediment (Fig 8). This group includes one sample from open-air site 1 (OA1-43 with 1.6M phytoliths/g sediment) collected from the middle part of the terrace trench; two samples from open-air site 2 (OA2-62 and OA2-76 with 1.3M and 1M /g, respectively) collected from the upper parts of two trench profiles (sq. OA2-D5 and E3) and samples taken from the eastern part of the rock-shelter (sq. RS-C3 and C7 north profile) (Fig 5 and S2 Table). These samples probably indicate areas of human activity associated with plant processing/use that have enriched the natural phytolith presence. The open-air site 2 and the rock-shelter samples were collected in proximity to the surface and therefore could also indicate phytolith deposition due to post-abandonment human activity [45, 54] and not necessarily be the direct result of primary *in situ* Nayaka activities. These samples could also represent specific locations of naturally occurring high phytolith concentrations. However, there are no differences in vegetation or taxa representation between the excavated sites and the spots sampled for control, and therefore we suggest that natural phytolith deposition is homogenous in all the study area. Thus, the accumulation of phytoliths with concentrations above 1M/g sediment should be regarded as in association to human activity.

The last group, with the highest concentrations of phytoliths of above 2M /g includes only one sample from the contemporary site (CS-101) and several samples from the western part of the rock-shelter (Figs 5 and 8). The ethnographic data and our field observations, coupled with the mineralogical and elemental analysis, suggest that the sample from the contemporary site (CS-101) is related to a hearth and the ashy remains of fuel. The relatively high phytolith concentration of 2.3M/g implies that ash remains preserve well even after clearing of the hearth, but probably only for a relatively short period of time. Indeed, other surface samples, from spots in which the presence of a hearth was supported by ethnographic, data did not show such high concentrations. Therefore, we suggest that repetitive sweeping and trampling of the terrace, together with the effect of precipitation, would eventually result in the complete dispersal of the ash remains. The samples from the open-air sites and the western part of the rock-shelter showing higher phytolith concentrations were more likely deposited in relation to other activities involving plant materials (e.g. presence of mats, baskets, construction materials etc.) and do not represent hearth signatures.

With five samples having more than 4M/g, the samples collected from the western part of the rock-shelter (in the upper and middle part of the profiles of sq. RS-C7—west profile only—and RS-B9) yielded the highest concentrations of phytoliths (Fig 5 and S2 Table). Deposits with such a high phytolith input have been clearly associated to human activities such as ash deposition [49, 52], stabling activity [50, 56], agricultural fields [51], storage [50, 54] and/or building materials [45]. The ethnographic data and the physical setting of the rock-shelter indicate that animal enclosures were not at the origin of these higher concentrations. In addition, as immediate-return hunter-gatherers (see [57] for definition of immediate and delay return systems among hunter-gatherers), the Nayaka do not store food or other plant materials [20]. The absence of calcite and the relatively low concentrations of P and Ba, when compared to the sample from the recent abandon hearth in the contemporary site (CS-101), rules out the possibility of being a very large fireplace or a sequence of them. Furthermore, while under the acidic conditions in the site wood ash in the form of calcite would dissolve, chemical residues and phytoliths will preserve [57, 58] and the ethnographic observations do not support the expected amount of fuel and size of a hearth suggested from such a deposition. In addition, the preservation of *in situ* hearths requires fast burial by colluvium [59]. The phytolith-rich samples from the western part of the rock-shelter were also collected from the top of the profile without a new colluvial sediment layer above. From an ethnographic point of view, the Nayaka are known to build a lean-to roof from grasses when settling in a rock-shelter, exploiting the natural rock as the back wall [3]. Nayaka generally do not recycle construction materials and these are often left to decay at the abandoned site. It is therefore possible that the phytolith-rich layer represents a collapsed lean-to thatch roof (e.g., [45]). The spatial distribution of the phytoliths over the western part of the rock-shelter, in addition to the high amount of phytoliths compared to the hearth sampled in the contemporary site, support such an interpretation.

Further study of the phytoliths assemblages including morphological analysis and identification of the taxonomy may in due course enhance the interpretation of the depositional processes of the phytoliths and their origins.

### Micromorphology

Thin sections were produced from undisturbed sample blocks collected from open-air site 1 house, terrace and slope, open-air site 2 terrace and from the western part of the rock-shelter (Fig 5). The key micromorphological features and their interpretations are presented in Table 1. Overall, most of the samples exhibited similar composition and structure with some variation between the various fabrics identified in each sample. Following Stoops’s [32] guidelines for this section description, all the samples had a clay-rich groundmass with a speckled b-fabric and an abundance ranging between 10–40%. Variation was also observed among the size and abundance of the coarse fraction, with some samples having only 5–30% silt to fine sand size grains, while others had grain sizes of silt to gravel with similar abundance. Generally, the mineralogy observed can be associated with the weathering of the local charnockite rock with quartz, hornblende, biotite, and plagioclase as major minerals (for detailed description of rocks petrology in the area see [18]). All the samples showed a porphyric c/f related distribution and variable bioturbation, caused mainly by various worms and insects.

**Table 1:** Description and interpretation of thin sections.

Sample	Location	Key micromorphological observations	Interpretation
MM_OA1-House	OA1—K1	1. Top fabric: Open crumbly, poorly sorted fabric with irregular aggregates, bioturbated, complex microstructure mainly with channels, chambers and vesicles voids	Advanced mud wall degradation
		2. Middle fabric: Poorly sorted fabric with irregular aggregates, bioturbated, clay coating with horizontal orientation, clay hypo-coatings, complex microstructure mainly with channels, chambers and vesicles voids	Initial mud wall degradation
		3. Pure clay layer: Two thin layers of pure oriented clay coating, dusty silty clay and humified organics above and between the clay layers and trampled into the layers	• House floor • Indication for cycles of washing and spreading mud on the house surface
		4. Low fabric: Moderately sorted fabric with fine sand-silty irregular aggregates, clay coating with horizontal orientation, clay hypo-coatings, complex microstructure mainly with channels, chambers and vesicles voids	Buried regional soil
MM_OA1-Terrace	OA1—F4	1. Top fabric: Blocky crumbly structure with irregular aggregates, heavily bioturbated, complex microstructure mainly with channels oriented in all directions	Recent post-abandonment colluvial deposits
		2. Middle fabric: similar to top fabric but the structure is better defined by vertical channels and smaller irregular aggregates. iron impregnation in the sediment and on rock fragments surface	Terrace surface
		3. Low fabric: Moderately sorted fabric with fine sand-silty aggregates, bioturbated, complex microstructure mainly with channels, chambers and vesicles voids	Buried regional soil
MM_OA1-Slope	OA1—G11	Fabric: Poorly sorted fabric with irregular aggregates, complex microstructure heavily bioturbated with small pellety aggregates due to soil fauna activity, high amount of microcharcoals, few partly dissolved bone fragments, disintegration of microcharcoals and iron impregnation	Waste area with indication for acidic conditions and slope deposition patterns
MM_OA2-Terrace	OA2—D5	1. Top fabric: Poorly sorted fabric with irregular aggregates, complex open microstructure, heavily bioturbated, high amount of organic matter, very small pellety aggregates due to soil fauna activity, replacement of organic matter with clay and iron, disintegration of microcharocals	Recent post-abandonment deposits mixed with possible terrace surface, indications for acidic conditions
		2. Low fabric: Similar to top fabric but more dense, more groundmass and weathered rock fragments	Possible terrace surface mixed with Bt horizon
MM_RS	RS—B9	1. Top fabric: Poorly sorted fabric with complex microstructure, heavily bioturbated, very small pellety aggregates due to soil fauna activity, high amount of organic matter	A horizon
		2. Middle fabric: Poorly sorted fabric with open porous complex structure, clumps of clay aggregates, fine rock fragments, few charcoals, heavily bioturbated with pellety aggregates due to soil fauna activity	Recent deposits with possible secondary post-abandonment human activity
		3. Organic layer: Mostly organic matter, secondary iron impregnation some silt and clay, ‘turf-like’ appearance	Collapsed thatch roof
		4. Middle fabric: similar to no. 2 but less porous and more compact, no charcoals and few rock fragments	Post-abandonment deposits
		5. Low fabric: Poorly sorted fabric with irregular aggregates, many coarse rock fragments, a thick charcoal horizon in the upper part of fabric, complex microstructure, bioturbation, ll the material is oriented with slope inclination	Possible in situ combustion feature laying within colluvial deposits from rock weathering

Each fabric/feature is numbered and ordered from top to bottom of the sample. OA1 = Open-air site 1; OA2 = Open-air site 2; RS = Rock-shelter

#### Open-air site 1 house

**Fig 9:**
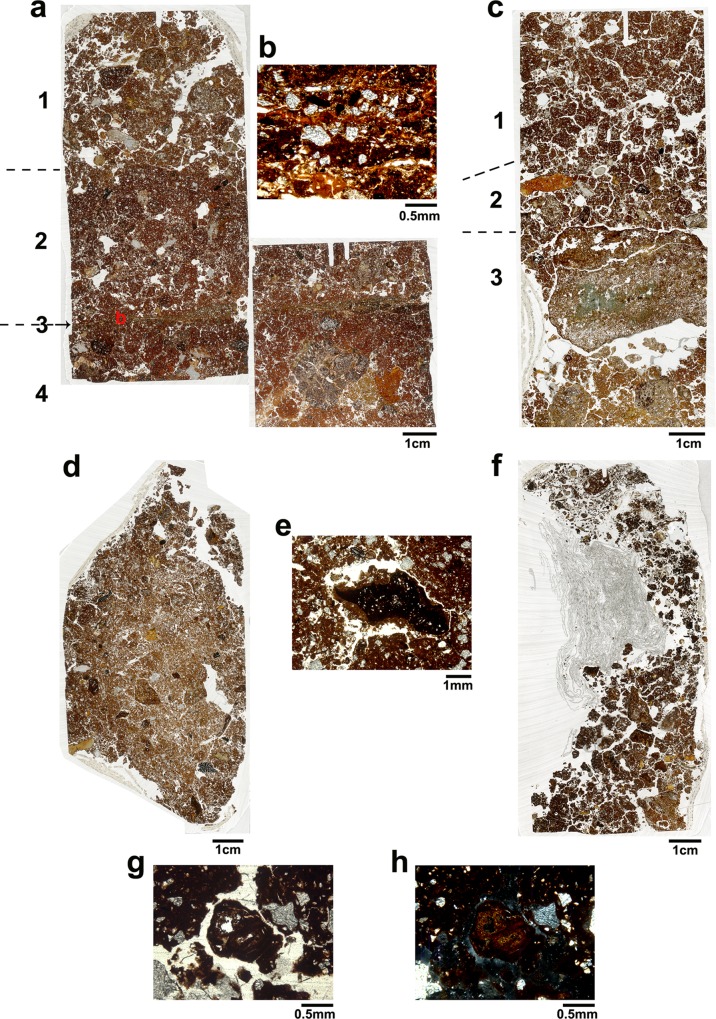
Key micromorphological features in the open-air sites. (a) Scan of MM_OA1-House showing two thin sections. The numbers indicate the key micromophological features described in Table 1. Note that no. ‘3’ is a thin horizon marked by the arrow. The letter ‘b’ indicates the location of the microphotograph; (b) Microphotograph of the horizon interpreted as the house floor showing two thin layers of pure oriented clay coating with dusty silty clay and humified organics above and between the clay layers. Note how quartz grains and organics are trampled into the clay layers. The photograph was taken in Plane Polarized Light (PPL); (c) Scan of MM_OA1-Terrace thin section. The numbers indicate the various fabrics identified and described in Table 1. Note the smaller size of the irregular aggregates and the horizontal orientation of the cracks in fabric 2 compared with fabric 1 where cracks are randomly oriented. Fabric 2 shows several past terrace surfaces. The rock fragment at the most top part of fabric 3 is showing iron impregnation on its surface indicating close proximity to the surface; (d) Scan of MM_OA1-Slope thin section. Fragments of well-preserved charcoals can be seen throughout the thin section. Note the orientation inclined with the slope towards the left side and the lack of clear layering; (e) A microphotgraph of a charcoal found in MM_OA1-Slope. The charcoal is covered by clay and yet show signs of disintegration. This taphonomic process indicate that while the charcoal is protected and covered by the local sediment, probably acidic conditions are affecting the charcoal and its preservation (PPL); (f) Scan of MM_OA2-Terrace thin section. The upper part of the sample show a crumbly structure and evidences to intensive biological activity which disrupt the sediment structure. Note the higher amount of organics in the upper part. The greyish material on the left is the remains of paper attached to the sediment as part of the sampling process; (g) Microphotograph showing the replacement of organic material with clay and secondary iron (PPL); (h) same microphotograph as ‘g’ (XPL).

The sample from open-air site 1 house (MM_OA1-House) was collected from the corner of the house near its wall, from the eastern profile of sq. OA1-K1 (Fig 5). Two thin sections samples were prepared and exhibited three different fabrics (Table 1). Whilst in terms of composition the three fabrics are rather similar, their structure suggests different deposition processes. The most upper part shows an open crumbly structure underlain by a denser fabric (no. 1 and 2 in Fig 9A). Yet, both fabrics exhibit a complex microstructure, evidence for bioturbation and indications of the gravitional deposition of material washed away by low energy flows. Thus, the two upper fabrics may be interpreted as degraded wall material accumulating within the abandoned house after being washed away from the wall (e.g., [55, 60]). Friesem et al. [60] has shown that during the initial phase of degradation of a mud structure, degraded mud-brick material is first accumulating on the floor near the base of the walls. As degradation continues more material is being washed away from the walls and redeposited within the decayed structure. Thus, on the floor near the walls degraded mud-brick material can be found in a better state of preservation [55, 60]. Therefore, the differences in density and bioturbation can be interpreted as resulting from initial degradation and redeposition of material, in contrast to more advanced degradation of the mud walls redepositing sediments in the upper fabric. In addition, the presence of clay hypo-coatings in the middle fabric suggest that the material used as raw material for the mud-bricks was collected from an argillic or Bt horizon (e.g., [55]). In comparison to the above fabrics, the lowermost fabric presents better sorting and smaller grain size. Also with clay hypo-coatings but without evidence for gravitational re-deposition of material, this fabric can either reflect the *in situ* buried soil that the house was built upon, or it may represent earthen construction material made from the local buried soil. In the latter case the structure of the fabric suggests that the material used as the floor make-up or for building the stage on which the house was built was not disturbed during the process. The similarity of the fabric to other fabrics found in the lowermost part of the profiles sampled elsewhere on the site strengthen its interpretation as the buried soil.

An horizon composed of two thin pure clay layers was found between the middle and low fabric (no. 3 in Fig 9A). This feature showed dusty silty clay and humified organic matter trampled into the pure clay layers (Fig 9B). The pure clay layers were probably formed as water flew over the surface at a low energy which could only wash away clay size particles and smear them over the surface. Such deposition pattern was observed ethnographically during routinely cleaning practices which involved people washing their house by spraying water on the floors and sweeping the earth floors with a broom, smearing a thin layer of clay. Thus, we interpret this layers, located between initial degradation mud walls from above and floor make-up or buried soil from below, as the house floor surface. The accumulation of dust and organics trampled into the spread mud floor further supports such an interpretation.

#### Open-air site 1 terrace

One thin section was produced from a sample collected from the eastern profile of sq. OA1-F4, in the trench through the terrace of open-air site 1 (Fig 5). The sample was divided into three different fabrics (Table 1). The lowermost fabric showed high similarity to the lowermost fabric in the house and was therefore interpreted as the local buried soil. Just above a large fragment of rock located within the low fabric, two other fabrics exhibited similar structure but with some differences. The uppermost fabric was more turbated due to biological activity and presented larger irregular aggregates compared to the irregular aggregate structure of the middle fabric (no. 2 in Fig 9C). In addition, while in the upper fabric channel voids were oriented in multiple directions, the crack orientation in the middle fabric was more vertical. Those features could suggest more stability in the sediment of the middle fabric and the formation of cracks as a result of repeated trampling (e.g., [61–64]). Associated with that level where high concentrations of phytoliths and heavy metals (Fig 5). Thus, the middle fabric as the terrace surface was where activity took place, while the top fabric is interpreted as recent post-abandonment deposition. In addition, the middle fabric has secondary iron impregnation in the forms of hypo-coatings which also appear in the upper crust of the rock fragment. Here the formation of secondary iron can be associated with the tropical forest environment [65, 66]. The process of iron oxide removal is favourable under oxygen depleted (anaerobic) water and where sufficient amount of organic matter is being decomposed by soil fauna [67]. The Indian tropical forests provide a fair amount of organic matter being intensively degraded by biological activity within the sediments. In addition, the sediments are saturated with water during the rainy seasons, especially in their parts close to the surface, followed by hot and dry conditions which favour the re-impregnation of the iron (e.g., [7–10]). The higher amount of secondary iron in the fabric 2 in the terrace may suggest more proximity to the surface.

#### Open-air site 1 slope

One sample was collected from the slope of open-air site 1 from the eastern profile of sq. OA1-G11 (Fig 5). The sediment of the slope did not exhibit any layering differences in the fabric (Fig 9D), but showed clear indications for slope deposition such as grains covered by continuous clay coatings formed during gravitational rolling of the grains down the slope. Grains were deposited with inclined orientation along the slope. A pellety structure was observed as a result of extensive biological activity within the sediment. The sample contained a large amount of charcoal of various sizes and states of preservation. Iron impregnation was found throughout the sample and a small fragment of bone exhibited dissolved edges. Pieces of charcoal covered by sediment showed signs of disintegration (Fig 9E). The dissolution of bone and ash suggest acidic conditions in the sediment. While the disintegration of charcoal material could be attributed to mechanical stress (e.g., trampling, sweeping, bioturbation) as charcoal tend to preserve well in acidic conditions [28] and tropical sites [7–10].

#### Open-air site 2 terrace

One sample was collected from open-air site 2 from the eastern profile of the trench in sq. OA2-D5 (Fig 5). According to the location of the wall remains, it is assumed that the sample represents the exterior terrace where activities took place. In addition, the block is associated with the sample containing relatively high amount of phytoliths (Fig 5 and S2 Table). The thin section made from this sample shows an open crumbly structure with evidence of significant biological activity, especially in the upper fabric (Table 1 and Fig 9F). The upper fabric has high amounts of organic matter, which in some cases is replaced by clay and iron (Fig 9G and 9H). Some micro-charcoal were also observed with signs of disintegration. In comparison to the top fabric, the low fabric has more groundmass and more weathered rock fragments. Based on the latter and the presence of micro-charcoal and organic matter in the top fabric, the low fabric is interpreted as the buried soil and the top fabric as the terrace surface mixed with recent post-abandonment deposits, both of which have been heavily bioturbated. The replacement of the organic matter with clay and iron and the disintegration of charcoal is consistent with the tropical and acidic conditions. Unfortunately the intensive biological activity in these superficial levels makes it impossible to clearly identify an occupation surface (e.g., [7, 8, 10]).

#### Rock-shelter

**Fig 10:**
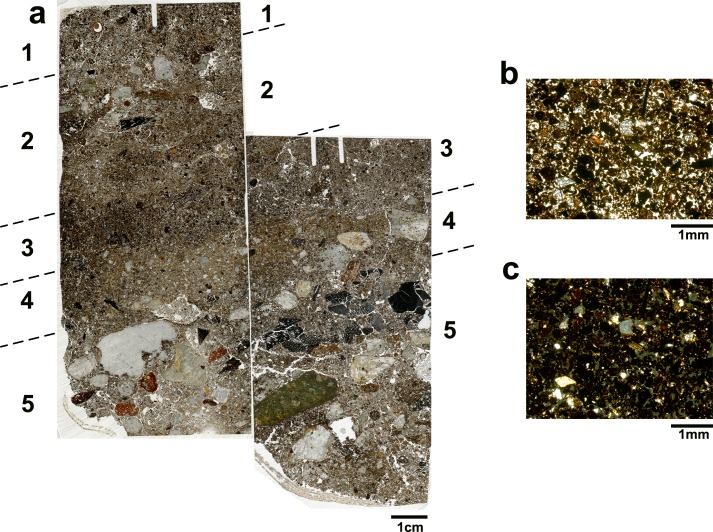
Key micromorphological features in the rock-shelter. (a) Scan of MM_RS showing two thin sections. The numbers indicate the various fabrics identified and described in Table 1. Note the inclination of the layers. Note that in fabric 2 the upper part contain more gravel than its lower part with a piece of charcoal in between the two parts. Also note the charcoals horizon in the upper part of fabric 5 also observed macroscopically in the field (Fig 4H); (b) Microphotogrpah of fabric 3, the ‘turf-like’ layer interpreted as the remains of a collapsed thatch lean-to roof. Note the abundance of organics within the crumbly matrix (PPL); (c) same microphotograph as ‘b’ only in XPL. Note: organics are opaque in XPL.

One block for micromorphological analysis was extracted from the southern profile of sq. RS-B9 in the rock-shelter (Fig 5). The sample was associated in the field with a thin charcoal layer (Fig 4F). Phytolith analysis showed high concentrations of phytoliths in the upper layers of the profile (Fig 5 and S2 Table), while elemental analysis indicated the relatively high concentrations of P, Ba and Mg (Fig 6A, 6C and 6D and S1 Table) which can be associated with deposition of organic matter. Overlapping thin sections were produced from the block which was divided into five different fabrics (Fig 10A and Table 1). The most upper layer, fabric 1, exhibits a poorly sorted fabric, heavily bioturbated with pellety aggregates due to soil fauna activity. The location of this fabric at the surface and the amount of degraded organic matter suggest the formation of an A horizon. Fabrics 2 and 4 were quite similar with an open crumbly structure showing clumps of clay aggregates along with sand size rock fragments. These fabrics were also heavily bioturbated with pellety aggregates due to soil fauna activity. While fabric 4 was less porous and had fewer rock fragments, fabric 2 contained some charcoal. Fabric 3 (between fabric 2 and 4) was mainly composed of organic matter with a ‘turf-like’ appearance (Fig 10B and 10C). Secondary iron impregnation and clay were also present within this layer. The lowermost layer, fabric 5, show a poorly sorted fabric with a complex microstructure, irregular aggregates with clear evidence for bioturbation. Fabric 5 included gravel size rock fragments, oriented according to the northward rock-shelter inclination. In addition, a horizon composed of well-preserved pieces of wood charcoal was observed between the gravels, also oriented according to the site inclination (Fig 10A).

Based on these observations, the lowermost fabric is interpreted as the surface on which activity took place, with the continuous deposition of gravels resulting from the rock weathering. The charcoal horizon represents an *in situ* hearth, coinciding with the site abandonment. Fabric 4 is interpreted as post-abandonment deposition of sediments and rock weathering, while the ‘turf-like’ fabric 3 represent the collapse of a lean-to thatched roof. This interpretation is further supported by the ethnographic data [3] and the high phytolith concentrations. Fabric 2, which is similar to fabric 4, is associated with post-abandonment deposition. The presence of charcoal suggests post-abandonment activity on top of the collapsed roof. The differences in the abundance of gravels within fabric 2 could be the result of localised events of rock weathering or post-abandonment activity. Finally, the uppermost fabric shows the most recent sediment accumulation and the formation of an A horizon, with the site no longer being active.

## Site Formation Processes

### Depositional processes related to hunter-gatherer activity

Nayaka architecture and their use of space reflects the importance of being-in-relation and living-together. In order to live together and maintain social relationships Nayaka people carry out most activities outdoors in full visibility of every person in the group, allowing a constant sharing of spaces, actions and things [3, 24]. Mobility and immediacy, two key social features among hunter-gatherers, are expressed not only by the movement of people but also through the ephemeral nature of their activity areas [23]. In terms of material deposition patterns, the Nayaka social dynamics dictate dynamic spatial deposition patterns, where no specific activity is confined to a specific space.

**Fig 11:**
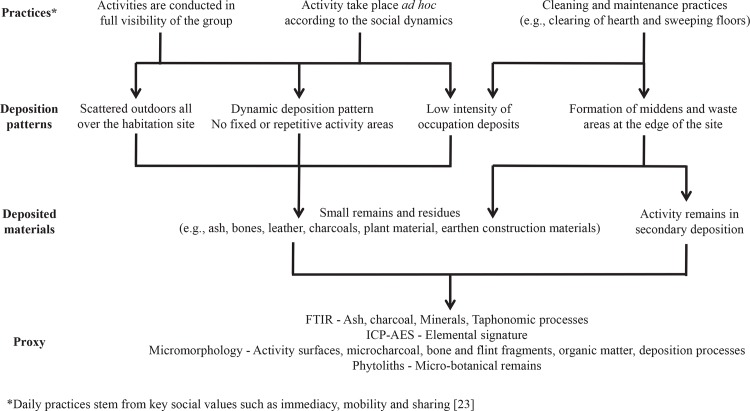
A model for material deposition patterns associated with hunter-gatherer activity and suggested proxies for their archaeological identification. ICP-AES = Inductively Coupled Plasma-Atomic Emission Spectrometry; FTIR = Fourier Transform Infrared Spectroscopy.

Nevertheless, while field observations from the abandoned sites could not identify macroscopic artefacts (beside some remains of houses following the clearing of the dense forest vegetation), laboratory-based analyses identified occupation surfaces and deposits associated with hunter-gatherer activity (Fig 11).

Using micromorphology analysis, the house floor in open-air site 1 was identified but the sediment analysis showed that within the houses the floor is clean from activity remains. The terraces of the open-air sites yielded some spots with relatively high concentrations of phytoliths and heavy elements. Nonetheless, the overall pattern of occupation deposits associated with human activity does not support well defined activity areas for the designated use of space. Rather, the microscopic and chemical analyses support the ethnographic observation of hunter-gatherers’ tendency to act outside on the terrace, and the ephemeral nature of their activity areas. Site maintenance and cleaning practices observed during the ethnographic work were also evident at the micro- and sub-microscopic scale. The house floor in open-air site 1 showed indications of washing off and cleaning, and while no charcoal was found anywhere within the house or the terrace, it was found in high concentrations in the slope sediments. It is therefore concluded that routine cleaning practices reduce the chances for the preservation of primary occupation deposits *in situ*, but enhance the chances for preserved horizons in secondary deposition on the slope beyond the living terrace used as the waste areas.

The ethnographic data described the rock-shelter as a site of human habitation of several families, living under the rock and grass lean-to roof for at least several months. Yet, the only macroscopic remains that could be defiantly associated with past human activity were a few charcoal fragments buried under the topsoil. When examining the site’s sediments through microscopic and chemical analysis it was possible to trace the activity surface and collapsed thatch roof. Overall, the rock-shelter presented much better preservation of activity remains than the open-air sites.

### Post-depositional processes related to tropical environments

**Fig 12:**
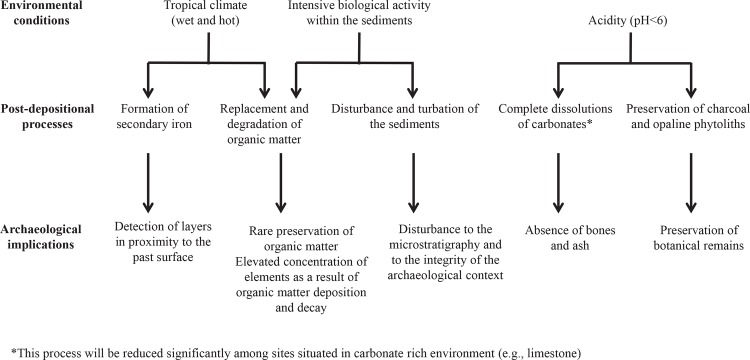
A model for post-depositional and taphonomic processes in tropical environments.

Archaeological site formation processes do not end with the deposition of materials due to human activity in the site. Post-depositional processes continue to affect the archaeological setting and may alter the archaeological record. The results of this study highlight the role of post-depositional and taphonomic processes in tropical forests and their implications for archaeological site formation processes in tropical environments (Fig 12).

Samples collected from the contemporary site were used as control for occupation deposits prior to post-depositional alteration. When comparing the contemporary site samples to those from the open-air and the rock-shelter sites, several altering factors become evident. The tropical sediments appear to exhibit acidic conditions which result in complete dissolution of carbonates such as wood ash (in the form of calcite) and bone (in the form of carbonate hydroxylapatite) (e.g., [7, 8, 10]). With pH values below 6, even after a few days to weeks of deposition, ash and bones will start to dissolve [28]. The formation of authigenic minerals such as carbonate fluorapatite is another indication of post-deposition processes probably associated with water rich in fluoride ions. From this research, it is evident that further study is required to understand the formation of authigenic mineral and diagenesis of archaeological materials in humid tropical environments [8].

The wet and warm conditions of tropical rainforests favor significant biological activity, which disrupts the sediment structure and enhances the degradation of organic matter. In addition, cycles of wet and dry conditions lead to impregnation with secondary iron [7, 8, 10]. The combination of soil acidity and faunal activity easily degrades the soil organic matter and in some cases replaces with iron and clay. All these processes prevent the good preservation of the deposit structure and of some components, making difficult the identification of occupation episodes and activity surfaces.

On the other hand, in acidic conditions charcoal and phytoliths do preserve and therefore can be used as markers for human activity in tropical forests. Being abandoned approximately at the same time, the rock-shelter presented a better state of preservation of organic matter as compared to the open-air sites. Yet, this could be the result of differences in the type of activity or due to the type of remains left behind during abandonment. Chemical analysis of elements associated with human activity (e.g. P, Sr, Ba, K, Na and Zn) were relatively high in the contemporary site, as expected, and lower in the rock-shelter and much lower in the open-air sites. These lower values could be associated with post-depositional processes which indicate that chemical markers for anthropogenic activity are better preserved in rock-shelters as compared to open-air sites.

## Conclusions

The ethnoarchaeological approach of this study was designed to identifying the formation processes of the archaeological record of hunter-gatherer groups in tropical moist forest and to understand the anthropogenic signatures preserved in the archaeological record. In the light of this study, we argue that this is possible only through the integration of macro-, micro- and sub-microscopic approaches, which are apt to untangling the depositional and post-depositional processes that created the archaeological record. The ephemeral and moveable activities were carried out outside the houses on the external terrace. Social practices, site maintenance, cleaning practices and the impact of monsoon rains meant that primary deposition is minimal with little spatial differentiation (there is no direct correlation between activity area and deposition). The waste deposits (middens) at the edge of the activity areas (edge of platforms) are the areas where most of the sedimentation takes place and is preserved. The moist tropical environment further complicates the depositional and post-depositional dynamics due to significant biological activity, which disrupt the sediment structure and degrade the organic matter. In addition, the acidic conditions of the forest soil result in the dissolution of wood ash and bone, but charcoal and phytoliths all be preserved nonetheless.

The current study has been able to identify the midden areas but also, and more importantly, highlighted the possibility of identifying the occupation surfaces and a few loci with primary deposition from *in situ* activities. Although the identification and reconstruction of hunter-gatherer activity in archaeological sites within tropical forests is challenging, the use of several microscopic and chemical analyses allows the identification of anthropogenic signatures. In turn, this enables the reconstruction of the site formation processes including human activities and the use of space as well as post-depositional processes taking place that alter and shape the archaeological record.

## Supporting Information

S1 Table Elemental analysis results using ICP-AES. OA1 = open air 1; OA2 = open-air 2; RS = rock-shelter; CS = contemporary site.
(XLSX)

S2 Table Phytolith analysis results. OA1 = open air 1; OA2 = open-air 2; RS = rock-shleter; CS = contemporary site.
(XLSX)
